# Persistent synovial inflammation plays important roles in persistent pain development in the rat knee before cartilage degradation reaches the subchondral bone

**DOI:** 10.1186/s12891-018-2221-5

**Published:** 2018-08-16

**Authors:** Takashi Hoshino, Kunikazu Tsuji, Hiroaki Onuma, Mio Udo, Hiroko Ueki, Masako Akiyama, Kahaer Abula, Hiroki Katagiri, Kazumasa Miyatake, Toshihumi Watanabe, Ichiro Sekiya, Hideyuki Koga, Takeshi Muneta

**Affiliations:** 1 Department of Joint Surgery and Sports Medicine, Graduate School, Tokyo Medical and Dental University, Tokyo, Japan; 2 Department of Cartilage Regeneration, Graduate School, Tokyo Medical and Dental University, 1-5-45 Yushima, Bunkyo-ku, Tokyo, 113-8510 Japan; 3 Research Administration Unit, Tokyo Medical and Dental university, Tokyo, Japan; 4 Center for Stem Cell and Regenerative Medicine, Tokyo Medical and Dental University, Tokyo, Japan

**Keywords:** Persistent pain, Monoiodo-acetic acid, Osteoarthritis model, CGRP

## Abstract

**Background:**

The major complaint of knee osteoarthritis (OA) is persistent pain. Unlike acute inflammatory pain, persistent pain is usually difficult to manage since its pathology is not fully understood. To elucidate the underlying mechanisms of persistent pain, we established 2 different inflammation-induced arthritis models by injecting monoiodo-acetic acid (MIA) into the joint cavity and performed integrated analyses of the structural changes in the synovial tissue and articular cartilage, sensory neuron rearrangement, and pain avoidance behavior in a rat arthritis model.

**Methods:**

Male Wistar rats received intra-articular injections of MIA (0.2 mg/30 μL, low-dose group; 1 mg/30 μL, high-dose group) in the right knee and phosphate buffered saline (PBS; 30 μL, control group) in the left knee. Fluorogold (FG), a retrograde neural tracer, was used to label the nerve fibers for the identification of sensory neurons that dominate the joints in the dorsal root ganglion (DRG). Both knees were subjected to the intra-articular injection of 2% FG in PBS (5 μL) under anesthesia 5–7 days prior to sacrifice. We performed pain avoidance behavior tests (incapacitance and von Frey tests) at 0, 1, 3, 5, 7, 14, 21, and 28 days. At 5, 14, and 28 days, the rats were sacrificed and the knee joint and DRG were excised for histological assessment. The knee joints were stained with hematoxylin and eosin, safranin O, and calcitonin gene-related peptide (CGRP). The DRG were immunostained with CGRP.

**Results:**

A transient inflammatory response followed by mild articular cartilage degeneration was observed in the low-dose MIA model versus persistent inflammation with structural changes in the synovial tissue (fibrosis) in the high-dose model. In the high-dose model, full-thickness cartilage degeneration was observed within 2 weeks post-MIA injection. The pain avoidance behavior tests indicated that persistent synovial inflammation and structural changes of the infrapatellar fat pad may play important roles in persistent knee joint pain before the articular cartilage degeneration reaches the subchondral bone.

**Conclusions:**

Transient inflammation without structural changes of the synovial tissues did not induce persistent pain in the rat knee joint before degradation of the articular cartilage reached the subchondral bone plate.

**Electronic supplementary material:**

The online version of this article (10.1186/s12891-018-2221-5) contains supplementary material, which is available to authorized users.

## Background

Osteoarthritis (OA) is a group of diseases and mechanical abnormalities involving degradation of the articular cartilage and subchondral bone [1]. The major complaint of OA patients is persistent knee pain, which significantly decreases their activities of daily living [2]. Therefore, most current treatment strategies for OA are based on symptoms by managing symptoms using anti-inflammatory analgesics and improving joint mobility and flexibility using programed exercise and weight control measures [3]. However, some patients develop uncontrolled persistent knee pain as the disease progresses.

The inflammatory response might play an important role in pain development in OA patients because topical nonsteroidal anti-inflammatory drugs are recommended as “appropriate” for all patients with knee-only OA by Osteoarthritis Research Society International (OARSI) guidelines [4]. However, it is unclear whether and to what degree OA-related persistent pain plays an important nociceptive role. Another important aspect related to persistent pain in OA is determining which components of the knee joint produce nociception. Baker et al. reported that synovitis is strongly related to maximal knee pain severity on the Western Ontario and McMaster Universities Osteoarthritis Index [5]. In a longitudinal study, Zhang et al. found that changes in bone marrow lesions (BMLs) and synovitis are associated with fluctuations in knee pain, and, most interestingly, pain resolution occurred more frequently when BMLs were smaller [6]. These data indicated that nociception of the synovial tissue and subchondral bone may play crucial roles in determining fluctuations in knee pain. However, the mechanisms of the development of persistent pain have not been elucidated. Furthermore, it is still unclear whether the nociceptive mechanism in persistent pain is the same as that in acute pain; if it differs, it is important to elucidate how persistent pain develops after acute inflammation has resolved. To answer to these questions, here we aimed to perform integrated analyses of the structural changes in synovial tissue and articular cartilage, sensory neuron rearrangement, and pain avoidance behavior in a monoiodo-acetic acid (MIA)–induced rat arthritis model.

The injection of MIA into the knee joint is an established and well-characterized animal model for OA [7–9]. The intra-articular injection of MIA induces synovial inflammation followed by articular cartilage degradation, a phenomenon that is consistent with human OA [10, 11]. Using MIA, we previously reported two different inflammation-induced articular cartilage degeneration models in rats [12]. One is to induce synovial inflammation by the injection of a relatively low dose (0.2 mg) of MIA. In this model, transient synovial inflammation was observed within 7 days, followed by the slow progression of articular cartilage degeneration by 28 days without obvious synovial inflammation after 14 days. The other is a high-dose (1.0 mg) injection model. In this model, the onset of acute inflammation is comparable to that of the low-dose model; however, synovial inflammation continues and structural changes consisting of synovial hyperplasia and fibrosis occur after 7 days. Articular cartilage degeneration reaching the subchondral bone is observed as soon as 14 days post-treatment. We consider the former the “transient inflammation followed by slow OA progression” model and the latter the “persistent inflammation with rapid cartilage degeneration” model. Using these two models, we analyzed the time course changes in pain avoidance behavior and compared them with the structural changes of the joint tissues and rearrangement of the sensory nerves, which are represented here by an increase in the density of calcitonin gene-related peptide (CGRP)–positive nerve fibers. Here we showed that low-dose MIA did not induce persistent pain instead of progressing articular cartilage degeneration. In contrast, persistent inflammation with structural changes induced continuous pain avoidance behavior throughout the experimental period (high-dose MIA). In these models, much denser CGRP-positive sensory nerve accumulation was observed in both synovial tissues and the L4 dorsal root ganglion (DRG). The findings observed in this study suggest that persistent inflammation, which induces irreversible structural changes to the synovial tissues, may play important roles in persistent pain.

## Method

### Materials

MIA, fluorogold (FG), and paraformaldehyde (PFA) were purchased from Sigma-Aldrich (St. Louis, MO, USA). Anti-human CGRP polyclonal antibody was purchased from Peninsula Laboratories LLC (San Carlos, CA, USA). Isoflurane, sucrose, and ethylenediaminetetraacetic acid (EDTA) were purchased from Wako Pure Chemical Industries Ltd. (Osaka, Japan). Mayer’s Hematoxylin and Eosin were purchased from Muto Pure Chemicals Inc. (Tokyo, Japan).

### MIA-induced arthritis model in rats

**Fig. 1:**
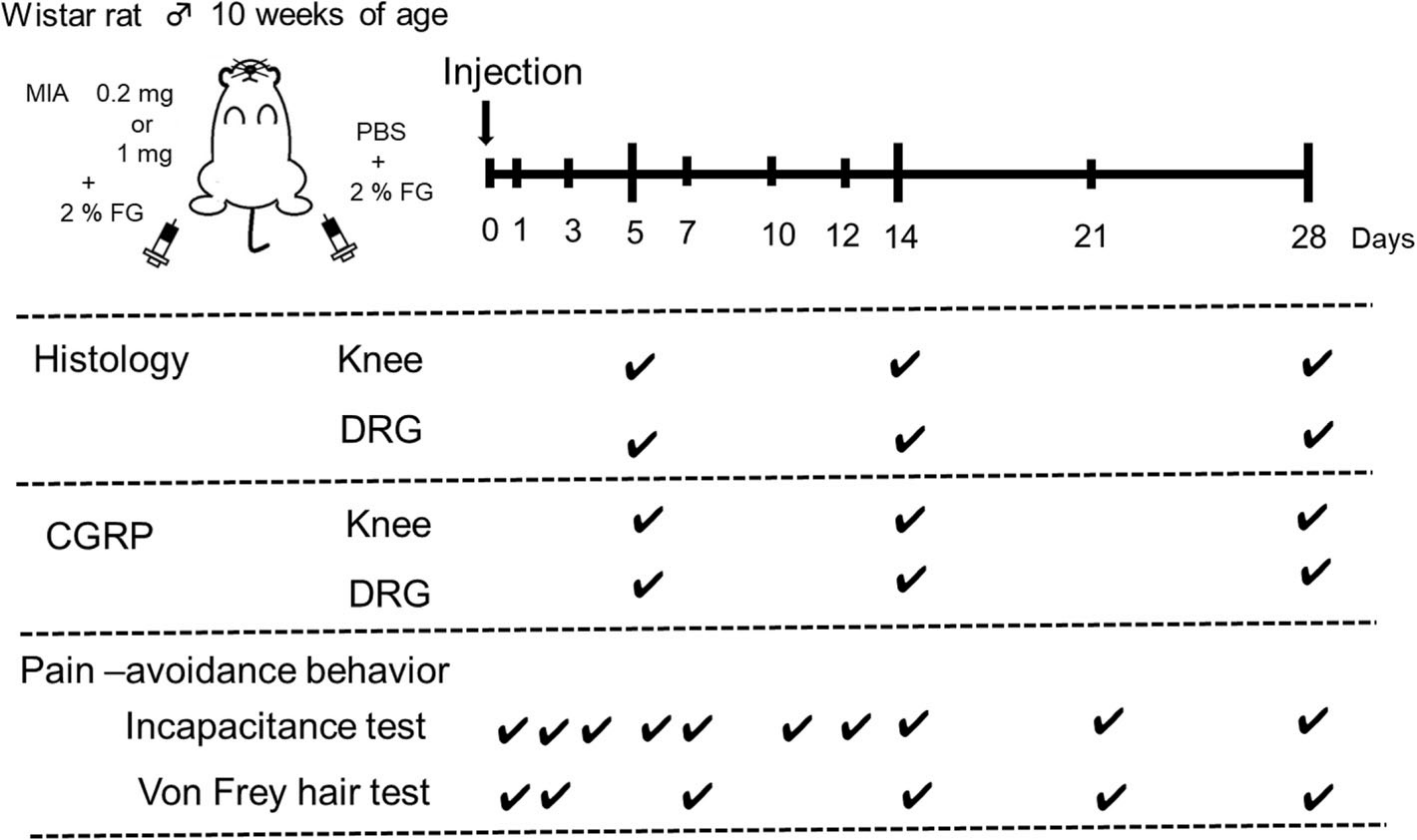
Study design. The right knee joint had an intra-articular injection of MIA at day 0. The left knee had PBS as a control. Histological and immunohistochemical evaluations (hematoxylin and eosin/safranin O staining and calcitonin gene-related peptide staining) were performed at 5, 14, and 28 days post-injection. The pain avoidance behavior tests (incapacitance and von Frey) were performed as indicated

The Institutional Animal Care and Use Committee of the Tokyo Medical and Dental University approved this study (approval no. A2017-259A). All animal experiments were conducted per the institutional guidelines. Thirty-six 10-week-old male Wistar rats (Charles River, Japan) weighing 330–345 g were used in this study. The rats were randomly divided into 2 groups (high-dose and low-dose). Rats were anesthetized by inhalation of isoflurane (2% in Oxygen, flow rate at 2 litters/min) before intra-articular injection. At day 0, the right knee joint was given an intra-articular injection of MIA in 30 μL of sterile saline as described previously [12]. The first 18 rats were given 1 mg of MIA, while the latter 18 rats were given 0.2 mg in the right knee joint. As experimental controls, 30 μL of phosphate buffered saline (PBS) was injected into the left knee joint on day 0. The rats were kept under a 12/12-h light/dark cycle with food and water ad libitum. At days 5, 14, and 28, they were sacrificed by perfusion fixation under deep anesthesia (inhalation of isoflurane, *n* = 6 at each time point), and both knee joints and DRG were excised for histological evaluation (Fig. 1).

### Retrograde nerve fiber labeling

Labeling of the nerve fibers was carried out using FG, a retrograde neural tracer, to identify sensory neurons that dominate the joints in DRG. Both knees were given an intra-articular injection of 2% FG in PBS (5 μL) under anesthesia (inhalation of isoflurane) 5–7 days prior to sacrifice [13, 14] (Fig. 1).

### Analyses of pain avoidance behavior

Weight-bearing asymmetry between the right (MIA side) and left (control side) limbs was assessed at day 0 (pre-injection), and at 1, 3, 5, 7, 10, 12, 14, 21, and 28 days post-injection (Fig. 1). The measurement was performed using an incapacitance tester (Linton Instrumentation, Norfolk, UK) [15, 16]. The rats were placed in a prismatic plexiglass case to allow the left and right hind limbs to be placed on an independent force plate. Each load amount was measured 100 times, and the percent weight of the ipsilateral hind limb was calculated according to the method described by Yu et al. [16].

The tactile hypersensitivity of the plantar of the hind paw was measured by von Frey hairs (Touch Test Sensory Evaluator, North Coast Medical Inc. Morgan Hill, CA, USA) according to the methods described by Orita et al. [13, 17]. In these experiments, the elasticity of the von Fray hairs was started at 300 g and gradually decreased until it reached 10 g. The maximum elasticity at which the rats did not express any escape behavior was recorded.

### Histological analysis

The rats were sacrificed at 5, 14, and 28 days after the intra-articular injection of MIA (Fig. 1). Under deep anesthesia (continuous inhalation of isoflurane), rats were transcardially perfused with PBS, followed by 500 mL of 4% paraformaldehyde (PFA). After fixation, both knee joints and spinal cords between T10 and S2 were excised. The knee joints were fixed with 4% PFA for 1 more week, then demineralized in 20% EDTA in PBS for 21 days and embedded in paraffin wax. The spinal cord was fixed in 4% PFA for 24 more hours and the L4 DRG was excised and dehydrated in a graded sucrose solution (7.5%, 15%, and 30%) to prepare frozen blocks.

To assess the severity of inflammation and structural changes of the infrapatellar fat pad (IFP), 5-μm-thick sagittal sections of the knee joint were prepared and stained with hematoxylin and eosin. Synovial tissue inflammation severity was semi-quantitatively evaluated using the IFP inflammation score according to the methods described previously (Additional file 1: Table S1) [12]. Severity of cartilage degeneration was evaluated using OARSI score (Additional file 2: Table S2) [18].

For the immunohistochemical staining of CGRP, frozen blocks of L4 DRG were sectioned in the axial direction at a thickness of 10 μm using Cryostat (CM3050 S, Leica Microsystems, Wetzlar, Germany). The sections were kept at room temperature for 30 min and incubated with rabbit anti-human CGRP antibody (1:400 dilution) at 4 °C for 20 h, rinsed with PBS 3 times, and incubated with Alexa 555-labeled goat anti-rabbit IgG antibody (Abcam, Carlsbad, CA, USA; 1:400 dilution) at room temperature for 1 h. The sections were rinsed with PBS 3 times and cover-slipped. Fluorescence images were captured using an Olympus BX53 microscope (Olympus, Tokyo, Japan).

To detect the CGRP-positive nerve fibers in the synovial tissues, the sections were de-paraffinized in xylene, rehydrated in graded alcohol, and rinsed with PBS. Subsequent incubations were performed in a humidified chamber. Endogenous peroxidases were quenched using 0.3% hydrogen peroxidase in methanol for 15 min. The sections were rinsed 3 times with PBS for 5 min, fixed again in 4% PFA, and briefly blocked with 10% normal goat serum (Vector Laboratories, Burlingame, CA, USA) to avoid non-specific antibody binding. The primary antibody for rabbit anti-human CGRP polyclonal antibody (1:250 dilution) was applied to the sections and incubated at 4 °C overnight. After the sections were rinsed with PBS 3 times, they were incubated in biotinylated goat anti-rabbit IgG secondary antibody (Vector Laboratories). Immunostaining was detected with the Vectastain ABC regent (Vector Laboratories) followed by diaminobenzidine staining. The sections were counterstained with hematoxylin. The innervation density of CGRP-positive nerve fibers was counted according to the methods reported by Tang et al. [19] and Mach et al. [20].

### Statistical analysis

The sample size was 6 in each group. The non-parametric Kruskal–Wallis test was performed, followed by the Steel-Dwass test, Mann-Whitney’s U test, Wilcoxon signed-rank test, and Dunnet test using SPSS software (v.24.0; SPSS, Chicago, IL, USA). *P* values less than 0.05 were considered significant.

## Results

### Injection of high-dose MIA into the knee joint induces structural changes in the synovial tissues and persistent pain in rats

Here we analyzed the time course of the pain avoidance behavior tests (incapacitance and von Frey) in high- and low-dose MIA models to examine if the differences in joint inflammation severity and duration causes different pain behavior in rats.

**Fig. 2:**
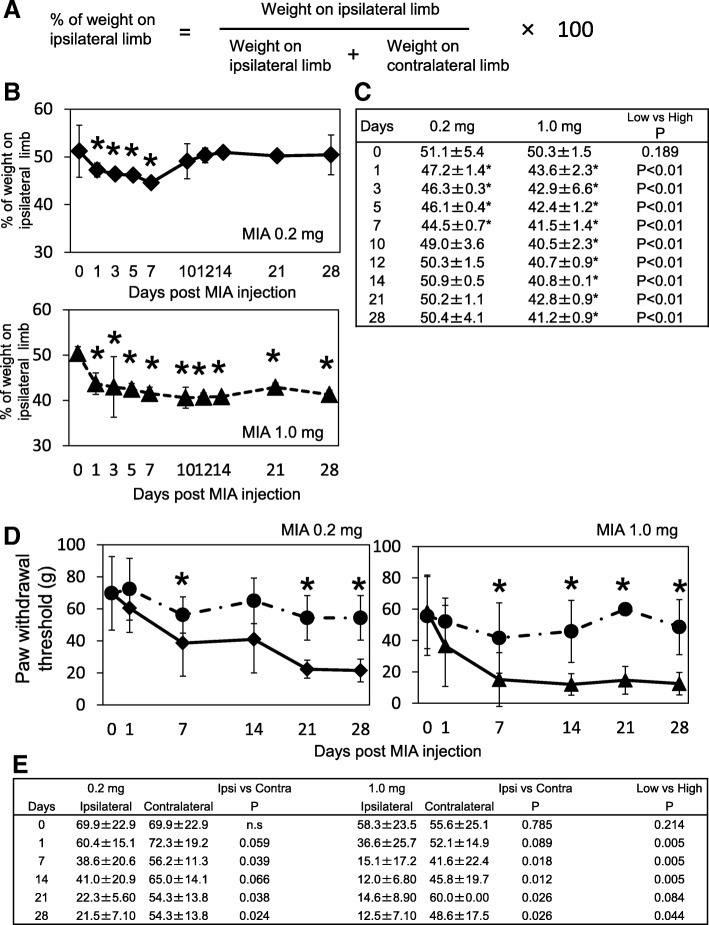
Pain avoidance behavior tests. **a** Incapacitance tests were performed and % of weight on the ipsilateral limb was calculated according to the indicated equation. **b** The time course changes of % weight on the ipsilateral limb was plotted. Asterisk indicated that the values were statistically significant compared to the pre-experimental values. There were 6 samples at each time point. **c** Mean and standard deviation values in panel B are indicated. Asterisks indicate that the values were statistically significant compared to pre-experimental values. The far right column indicated the *p* values between the low- and high-dose groups. **d** Time course changes of paw withdrawal threshold measured using von Frey hairs. Asterisks indicate that the values were statistically significant between ipsilateral side and contralateral side. There were 6 samples in each time point. **e** Mean and SD values in panel D are indicated. Asterisks indicate that the values were statistically significant compared the pre-experimental values (day 0). The 4th and 6th columns indicate the p values of the ipsilateral and contralateral limbs. The far right column indicates the p values of the low- and high-dose group at each time point

Figure 2 describes the results of the incapacitance tests. The percent of weight on the ipsilateral limb (Fig. 2a) gradually decreased by day 7 post-MIA injection in the low-dose group. The minimum load sharing ratio on the ipsilateral hind limb was 44.5 ± 0.7% in the low-dose group at day 7 post-MIA injection (Fig. 2b, c). In the high-dose group, weight-bearing reduced much quicker than that of the low-dose group and the minimum load sharing ratio on the ipsilateral hind limb was reduced to 40.5 ± 2.3% at day 10 (Fig 2b, c). The time course after reaching the minimum load sharing ratio was completely different between groups. In the low-dose group, the pain avoidance behavior gradually reversed and returned to pre-experimental levels by day 10 post-injection and never decreased again throughout the experimental period (Fig. 2b). In contrast, the load sharing ratio continued to decrease at the same level with the minimum ratio throughout the experimental period in the high-dose group after 10 days (Fig. 2b).

Tactile hypersensitivity of the plantar of the hind paw was measured using von Frey hairs (Fig. 2d, e). In contrast to the results of incapacitance test, the paw withdrawal threshold was gradually decreased until day 7 after the MIA injection and continued to decrease throughout the experimental period in both groups (Fig. 2d). The minimal withdrawal threshold did not seem different compared to the amount of MIA injected at days 21 and 28 (Fig. 2e).

**Fig. 3:**
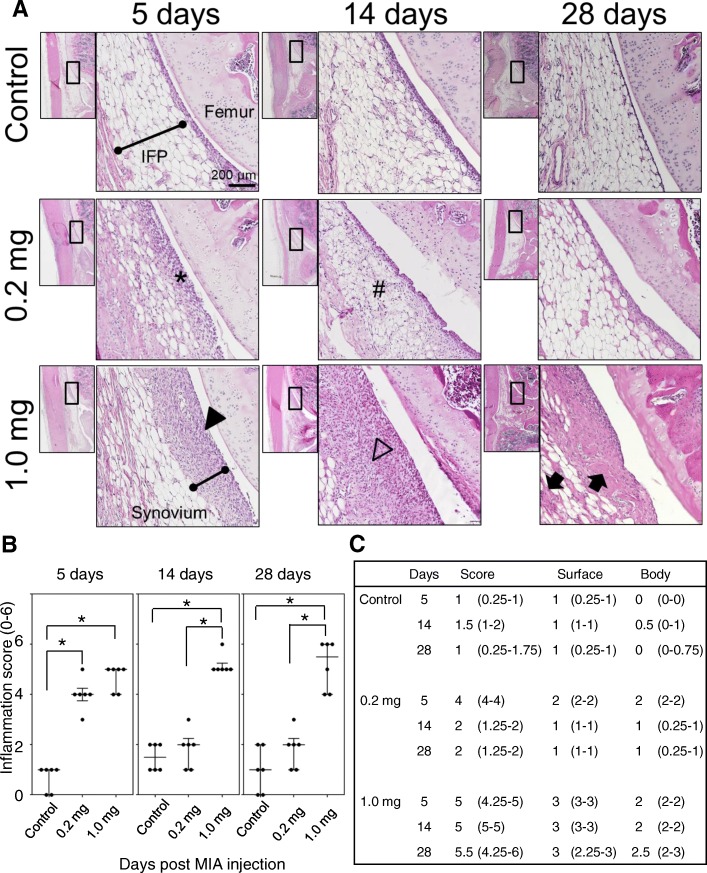
Inflammatory response of the synovial membrane and infrapatellar fat pad after the monoiodo-acetic acid injection. **a** Representative images of hematoxylin and eosin staining of sagittal sections of synovial tissues at each time point. The area indicated in the black box in low magnification image is enlarged and indicated. **b**, **c** Infrapatellar fat pad inflammation score was blindly evaluated by two independent researchers and data are presented in these panels. There were 6 samples at each time point. Four sections were randomly selected from each sample and evaluated. The median values and quartiles were recorded. The asterisks indicate the statistically significant values

Since the incapacitance tests suggested the sign of persistent pain in the high-dose MIA group, we performed detailed histological analyses to understand the underlying mechanisms of the pain persistence. The cellularity and structural changes of the synovial tissues were assessed by the hypercellularity of the synovial membrane located at the IFP surface and the structural changes (cellularity and fibrosis) as described previously [12]. In the low-dose group, the hyperplastic changes in the synovial membrane were observed within 5 days post-MIA injection (Fig. 3a, indicated by *). At day 14, synovial membrane cellularity seemed to decrease, while we observed mild inflammatory cell migration in the IFP body (Fig. 3a, indicated by #). However, these histological changes were reversible, and no significant structural alterations in the synovial membrane and IFP body were observed at day 28 in the low-dose group (Fig. 3a). The onset of the synovial inflammatory response was almost comparable between the high- and low-dose groups (Fig. 3a, b). Hyperplastic changes of the synovial membrane were also observed at 5 days post-MIA injection (Fig. 3a arrowhead). In contrast to the low-dose group, these hyperplastic changes in the synovial membrane were never alleviated with time in the high-dose group. Rather, the synovial hyperplasia was exacerbated after day 5, which extensively invaded the IFP body at day 14 (Fig. 3a, indicated by the open arrowhead). Cellularity in the synovial membrane and IFP body seemed lower at day 28 (Fig. 3a); however, the histological observation indicated the accumulation of extra-cellular matrices in the IFP body (Fig. 3a, indicated by closed arrow). These histological observations were semi-quantitatively evaluated by the scoring system described by Udo et al. (Additional file 1: Table S1) [12]. As shown in Fig. 3b and c, the onset of synovial inflammation was quite comparable regardless of MIA injection amount; however, it was quickly alleviated to the control levels by day 14 in the low-dose group (Fig. 3b, c).

**Fig. 4:**
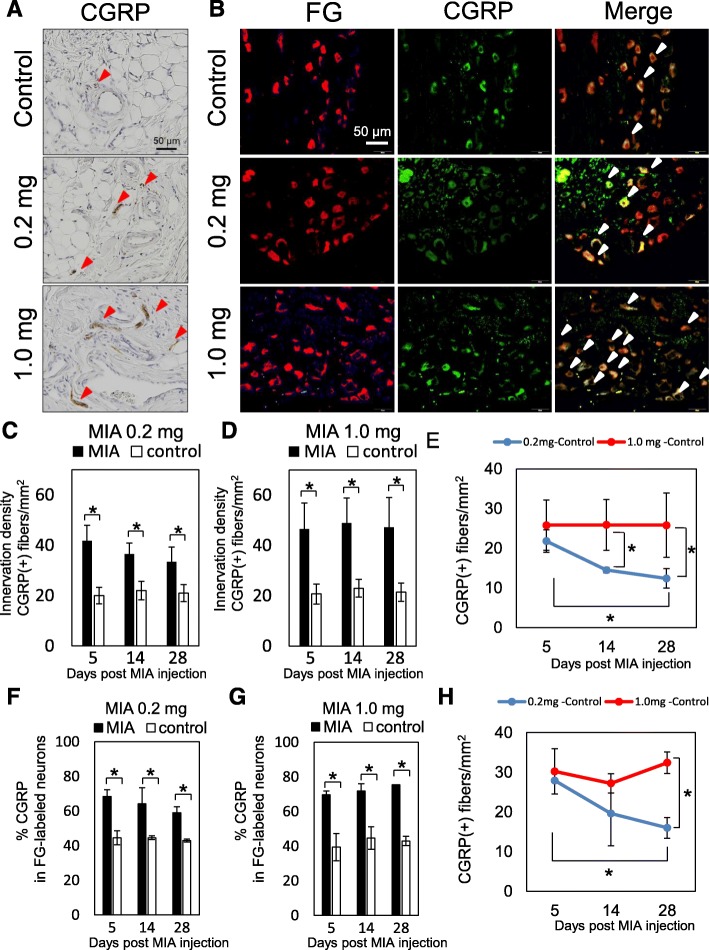
Distribution of CGRP-positive nerve fibers in IFP and L4 DRG. **a** Representative immunohistochemical images of the knee joint at 28 days after the injection of MIA. Arrowheads in red indicate CGRP-positive nerve fibers. **b** Representative images of L4 DRG at day 28 post-MIA injection. The red signal indicates neuronal cell bodies projected from the knee joint (left column, FG). Neural cells positive for CGRP are indicated in green (middle column). Merged images are shown in the right column. Arrows in white indicate the CGRP-positive nerve cells projected from the knee joint. **c**, **d** Differences in innervation density between the MIA and control sides. Ten different areas of 0.01 mm^2^ were randomly selected in the parenchymal region of IFP in each section. CGRP-positive nerve fibers > 0.03 mm were counted. There were 6 samples at each time point and 2 sections were randomly selected in each sample. Data are represented as mean and SD values. **e** Differences in innervation density between ipsilateral and contralateral indicated in (**c**) and (**d**) were calculated in each time point (innervation density of MIA subtracted by innervation density of control) and plotted. **f**, **g** The percentage of CGRP-positive neurons among the FG-labeled neurons. There were 6 samples in each time point. Four sections were randomly selected from each sample and the mean ± SD values were recorded (*n* = 6). Asterisks indicate statistically significant differences. **h** Differences in innervation density between ipsilateral and contralateral indicated in (**f**) and (**g**) were calculated in each time point (innervation density of MIA subtracted by innervation density of control) and plotted. CGRP, calcitonin gene-related peptide; DRG, dorsal root ganglion; FG, fluorogold; IFP, infrapatellar fat pad; MIA, monoiodo-acetic acid

To examine if these structural changes in the synovial tissues play important roles in nociception in rats, the distribution of CGRP-expressing sensory nerve fibers in the IFP body and L4 DRG were assessed by immunohistochemical staining (Fig. 4). As shown in Fig. 4a, b, c, and d, significantly higher numbers of CGRP-positive nerve fibers were observed in the IFP body after MIA injection compared to that of the contralateral side in both groups. In the low-dose group, CGRP-positive nerve fibers in the ipsilateral IFP body were significantly decreased at day 28 post-MIA injection (Fig. 4e), although it was still significantly higher than that of the contralateral side (Fig. 4c). In the high-dose group, CGRP-positive nerve fibers were increased compared to that of low-dose group and did not decrease at day 28 (Fig. 4d and e). Similar results were observed in the L4 DRG (Fig. 4f, g, and h).

### Time course changes of articular cartilage degradation after MIA injection

**Fig. 5:**
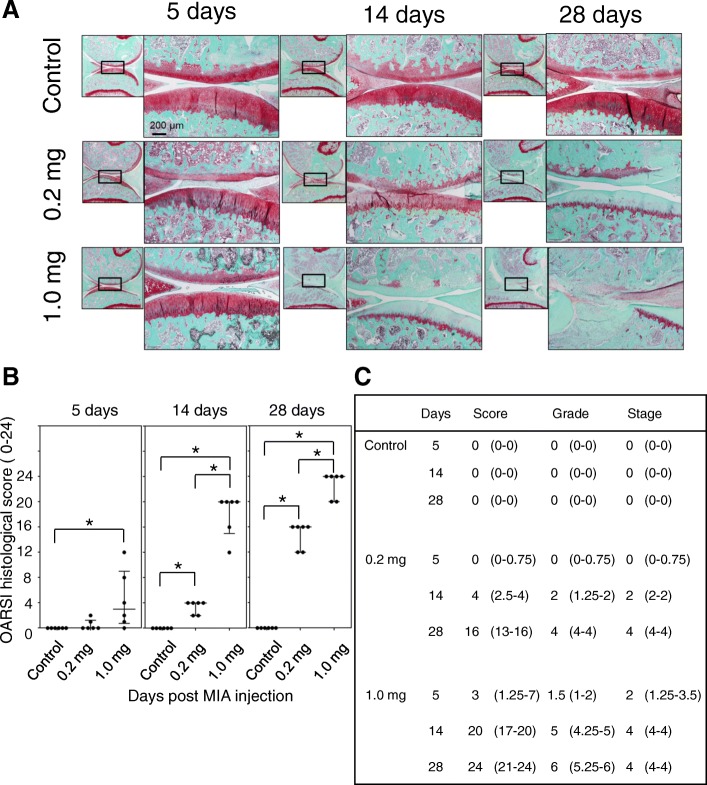
Articular cartilage changes after the MIA injection. **a** Representative images of Safranin-O staining of sagittal sections of the medial femoral and tibial condyles at each time point. **b**, **c** Osteoarthritis Research Society International histological scores were blindly evaluated by two independent researchers and data are presented in these panels. There were 6 samples at each time point. Four sections were randomly selected from each sample and the mean values were recorded. Median and quartile values in panel C are indicated. Asterisks indicate statistically significant differences. MIA, monoiodo-acetic acid

Since sensory nerve fibers do not exist in the articular cartilage, nociception does not seem to occur until degradation of the articular cartilage reaches the subchondral bone plate. Several reports have indicated that inflammatory signals enhance catabolic processes by inducing several cartilage-degrading enzymes such as matrix metalloproteins 1, 3, and 13 [21, 22]. Furthermore, inhibitors of these enzymes reportedly alleviate joint pain in several animal OA models [22]. To examine the time course changes in articular cartilage degradation in both experimental conditions, we performed a histological assessment of articular cartilage in accordance using the method described previously (OARSI scoring system, Additional file 2: Table S2 and Fig. 5) [18]. As shown in Fig. 5a, b, and c, the articular cartilage degradation gradually progressed with time after MIA injection in the low-dose group. As shown in Fig. 5c, the semi-quantitative OARSI scoring system indicated that degradation of the articular cartilage had not reached the subchondral bone even at day 28 after MIA injection (OARSI grades: rat 1 = 4; rat 2 = 4; rat 3 = 4; rat 4 = 3; rat 5 = 4; rat 6 = 4; Additional file 2: Table S2), and we did not observe a significant reduction in pain avoidance behavior in this group after day 14 (Fig. 2b). The articular cartilage degradation progressed much quicker in the high-dose group (Fig. 5a, b, c). Degradation reaching the subchondral bone was observed in 4 of 6 rats at day 14 post-MIA injection (OARSI grades: rat 1 = 3; rat 2 = 5; rat 3 = 4; rat 4 = 5; rat 5 = 5; rat 6 = 5) and most of the cartilage matrix was gone in almost the entire region of the articular surface at day 28 (Fig. 5c; OARSI grade: rat 1 = 6; rat 2 = 6; rat 3 = 5; rat 4 = 6; rat 5 = 5; rat 6 = 6).

## Discussion

In this study, we analyzed the time course changes in pain avoidance behavior in two distinct rat MIA-induced joint inflammation models. We showed that the intra-articular injection of low-dose MIA, which induced transient inflammation without inducing irreversible structural changes in synovial tissues, did not induce persistent pain in rats, while high-dose MIA, in which persistent inflammatory responses and fibrotic changes were observed in IFP, induced it throughout the experimental period. These data suggest that irreversible structural changes in synovial tissues after acute inflammation may play important roles in persistent joint pain. We expect that these two different joint inflammation models will be good tools to elucidate the molecular and cellular mechanisms of persistent joint pain.

Previous reports suggested that the major components of the knee joint that produce nociception are synovial tissue and epiphyseal bone marrow [23, 24]. In contrast, nociceptors do not exist in articular cartilage itself [25]. These data led us to consider that subchondral bone and bone marrow may not be the primary receptors for nociception in the early stage of OA, in which joint degeneration is limited within articular cartilage and has not reached the subchondral bone plate. Our data support this idea because the rats in the low-dose group did not seem to have joint pain after 14 days (Fig. 2b). In this experimental condition, we observed a reciprocal correlation between synovial inflammation severity (Fig. 3b) and pain avoidance behavior (Fig. 2b) from 0 to 14 days post-MIA injection; i.e. the minimum load sharing ratio on the ipsilateral hind limb decreased as the synovial inflammation progressed until day 7 post-MIA injection and then ratio returned to the pre-experiment level as the synovial inflammation was alleviated. These findings suggest a direct correlation between synovial inflammation and joint pain. Regarding articular cartilage degradation, the OARSI scoring system indicated that the articular cartilage degradation grade was 2 (range, 1.25–2) at day 14 and 4 (range, 4–4) at day 28 post-MIA injection, indicating that the tissue degradation did not reach the subchondral bone plate throughout the experimental period in the low-dose group (Fig. 5c, Additional file 2: Table S2).

In the high-dose group, the rats seemed to have persistent pain after day 7 (Fig. 2b). The histological assessments indicated that acute synovial inflammation occurred within 5 days post-MIA injection and continued throughout the experimental period (Fig. 3b). In this experimental condition, we also observed a reciprocal correlation between synovial inflammation severity and pain avoidance behavior (Figs. 2b, 3b), suggesting the importance of persistent synovial inflammation on the establishment of persistent joint pain. However, we observed that the full-thickness articular cartilage degradation reached the surface of the subchondral bone at day 14 post-MIA-injection in this group (OARSI grade of 5 [range, 4.25–5] at day 14 and 6 [range, 5.25–6] at day 28; Fig. 5c). Thus, it remains unclear if persistent inflammation in the synovial membrane and IFP is necessary and sufficient to establish persistent joint pain. Yu et al. reported that inhibition of the subchondral bone lesion by bone modifying drugs (zoledronic acid) significantly relieved joint pain in rat MIA-induced joint inflammation models [16]. To further understand persistent joint pain development in detail, we consider it necessary to establish the other experimental model in which rats have persistent synovial inflammation without articular cartilage degeneration. This project is among our next experimental plans.

CGRP is a 37-amino-acid pain–related neuropeptide arising from the DRG [26]. CGRP is expressed in human and rodent DRG neurons and knee joint tissues [27–30]. Molecular analyses indicated that the peripheral release of CGRP contributes to the vasodilation of acute neurogenic inflammation [31]. CGRP also reportedly functions as a component of molecular pathways that include other neuropeptides such as Substance P, TRPV1 ion channels, and tropomyosin receptor kinase-A (TrkA), a receptor for nerve growth factor [32]. Thus, the physiological functions of CGRP in the joint might contribute to both inflammation and joint afferent sensitization. In this study, we compared the distribution of CGRP-positive sensory nerve fibers between the low- and high-dose groups to understand the roles of CGRP in the development of persistent joint pain. The distribution of CGRP-positive fibers in the DRG and synovium was significantly higher in the MIA groups than in the control group throughout the experimental period (Fig. 4c, d, f, g; open and closed bars) and much higher in the high-dose group (Fig. 4e, h; 0.2 mg and 1.0 mg treatments). In the low-dose group, the distribution of CGRP-positive fibers was significantly decreased at day 28 versus day 5 (Fig. 4e, h). In contrast, the distribution of CGRP-positive fibers at day 28 was comparable to that of day 5 (Fig. 4e, h) in the high-dose group. These findings suggest that the continuous upregulation of CGRP in the nerve fibers may play some roles in persistent pain development. Although we did not check the time course changes in TrkA and TRPV1 expression levels in these models, it is feasible that these levels may be upregulated during persistent pain development. It is interesting to note that the distribution of CGRP-positive fibers was still significantly higher than that of the control in the low-dose group at day 28, when the weight-bearing ratio had already returned to the pre-experimental levels. These findings suggest that the quenching of persistent CGRP expression after synovial inflammation may have had functions rather than persistent pain development. We expect that persistent CGRP expression may play roles in controlling the pain threshold by maintaining the expression levels of receptors for other neuropeptides, such as TrkA and TRPV1, in a ligand-independent manner. Further studies are needed to test this hypothesis.

## Conclusions

In conclusion, here we showed that transient inflammation without structural IFP changes did not induce persistent pain in the knee joint before the cartilage degradation reached the subchondral bone. We expect that these two different joint inflammation models can be good tools to elucidate the molecular and cellular mechanisms of persistent joint pain development.

## Additional files

Additional file 1: **Table S1.** Grading scheme for assessment of infrapatellar fat pad (IFP) inflammation. This IFP inflammation grading scheme consists of “Cell infiltration at the surface of the IFP” and “Fibrosis in the body of the IFP” [12]. (DOCX 32 kb)

Additional file 2: **Table S2.** Osteoarthritis cartilage histopathology: grading and staging. An OA cartilage pathology assessment system deliberated by OARSI Working Group [18]. (DOCX 32 kb)

## Abbreviations

BML: Bone marrow lesion
CGRP: Calcitonin gene-related peptide
DRG: Dorsal root ganglion
EDTA: Ethylenediaminetetraacetic acid
FG: Fluorogold
HE: Hematoxylin and eosin
IFP: Infrapatellar fat pad
MIA: Monoiodo-acetic acid
OA: Osteoarthritis
OARSI: Osteoarthritis Research Society International
PBS: Phosphate buffered saline
PFA: Paraformaldehyde
TrkA: Tropomyosin receptor kinase-A
WOMAC: Western Ontario and McMaster Universities Osteoarthritis Index
