# Supplementary material for: Persistent synovial inflammation plays important roles in persistent pain development in the rat knee before cartilage degradation reaches the subchondral bone
Source: BMC Musculoskelet Disord. 2018 Aug 16;19:291. doi: 10.1186/s12891-018-2221-5 (PMC6097215; doi:10.1186/s12891-018-2221-5)
Supplement: Supplementary file 1 — Table S1. Grading scheme for assessment of infrapatellar fat pad (IFP) inflammation. This IFP inflammation grading scheme consists of “Cell infiltration at the surface of the IFP” and “Fibrosis in the body of the IFP” [12]. (DOCX 32 kb) [file 12891_2018_2221_MOESM1_ESM.docx]

Additional file 1

Supplemental Table 1. Grading scheme for assessment of infrapatellar fat pad (IFP) inflammation.

This IFP inflammation grading scheme consists of “Cell infiltration at the surface of the IFP” and “Fibrosis in the body of the IFP” [12].

| Cell infiltration at the surface of the IFP | |
| --- | --- |
| Points | Histological signs |
| 0 | Normal |
| 1 | Cellularity is increased, multinucleated cells present |
| 2 | Thickened lining cells, low （＜threefold thickness of the normal synovium） |
| 3 | Thickened lining cells, high （＞threefold thickness of the normal synovium） |
|  |  |
| Fibrosis in the body of the IFP | |
| Points | Histological signs |
| 0 | No fibrotic lesion |
| 1 | Fibrotic lesion in infrapatellar fat-pad present, low |
| 2 | Fibrotic lesion is increased, high |
| 3 | Infrapatellar fat-pad filled with the fibrotic lesion and fat cells absent |
