# Supplementary material for: Persistent synovial inflammation plays important roles in persistent pain development in the rat knee before cartilage degradation reaches the subchondral bone
Source: BMC Musculoskelet Disord. 2018 Aug 16;19:291. doi: 10.1186/s12891-018-2221-5 (PMC6097215; doi:10.1186/s12891-018-2221-5)
Supplement: Supplementary file 2 — Table S2. Osteoarthritis cartilage histopathology: grading and staging. An OA cartilage pathology assessment system deliberated by OARSI Working Group [18]. (DOCX 32 kb) [file 12891_2018_2221_MOESM2_ESM.docx]

Additional file 2

Supplemental Table 2. Osteoarthritis cartilage histopathology: grading and staging.

An OA cartilage pathology assessment system deliberated by OARSI Working Group [18].

| Grade |  | Stage | % Involvement |
| --- | --- | --- | --- |
| Grade 0 | surface intact  cartilage intact | Stage 0 | No OA activity seen |
| Grade 1 | surface intact | Stage 1 | <10% |
| Grade 2 | surface discontinuity | Stage 2 | 10-25% |
| Grade 3 | vertical fissures | Stage 3 | 25-50% |
| Grade 4 | erosion | Stage 4 | >50% |
| Grade 5 | denudation |  |  |
| Grade 6 | deformation |  |  |
